# Ventricular Septal Rupture in Cardiac Sarcoidosis Diagnosed by Point-of-Care Ultrasound

**DOI:** 10.7759/cureus.81261

**Published:** 2025-03-26

**Authors:** Christopher L Wilson, Melissa Edwards, Christopher Brown, Gowtham Grandhi

**Affiliations:** 1 Emergency Medicine, Virginia Commonwealth University, Richmond, USA; 2 Cardiology, Virginia Commonwealth University, Richmond, USA

**Keywords:** point-of-care-ultrasound, sarcoidosis, septal rupture, ventricular septal defect (vsd), ventricular septal rupture

## Abstract

A 60-year-old female with cardiac sarcoidosis and a history of ventricular aneurysm presented with acute palpitations and diaphoresis. Bedside point-of-care ultrasound (POCUS) identified a ventricular septal rupture (VSR) with left-to-right shunting, suggesting acute rupture of the aneurysm. This case highlights the critical role of POCUS in a rare and life-threatening complication of cardiac sarcoidosis, expediting management and improving patient outcomes.

## Introduction

Sarcoidosis is a complex chronic inflammatory disease with a tendency for multiple organ involvement including lungs, skin, eyes, and in this case, heart [1]. Cardiac sarcoidosis is a known cause of ventricular septal thinning and aneurysm formation; this case demonstrates ventricular septal rupture (VSR) as a very rare and life-threatening complication of this disease that was initially identified by bedside point-of-care ultrasound (POCUS) in a resource-limited emergency department [2].

## Case presentation

**Figure 1:**
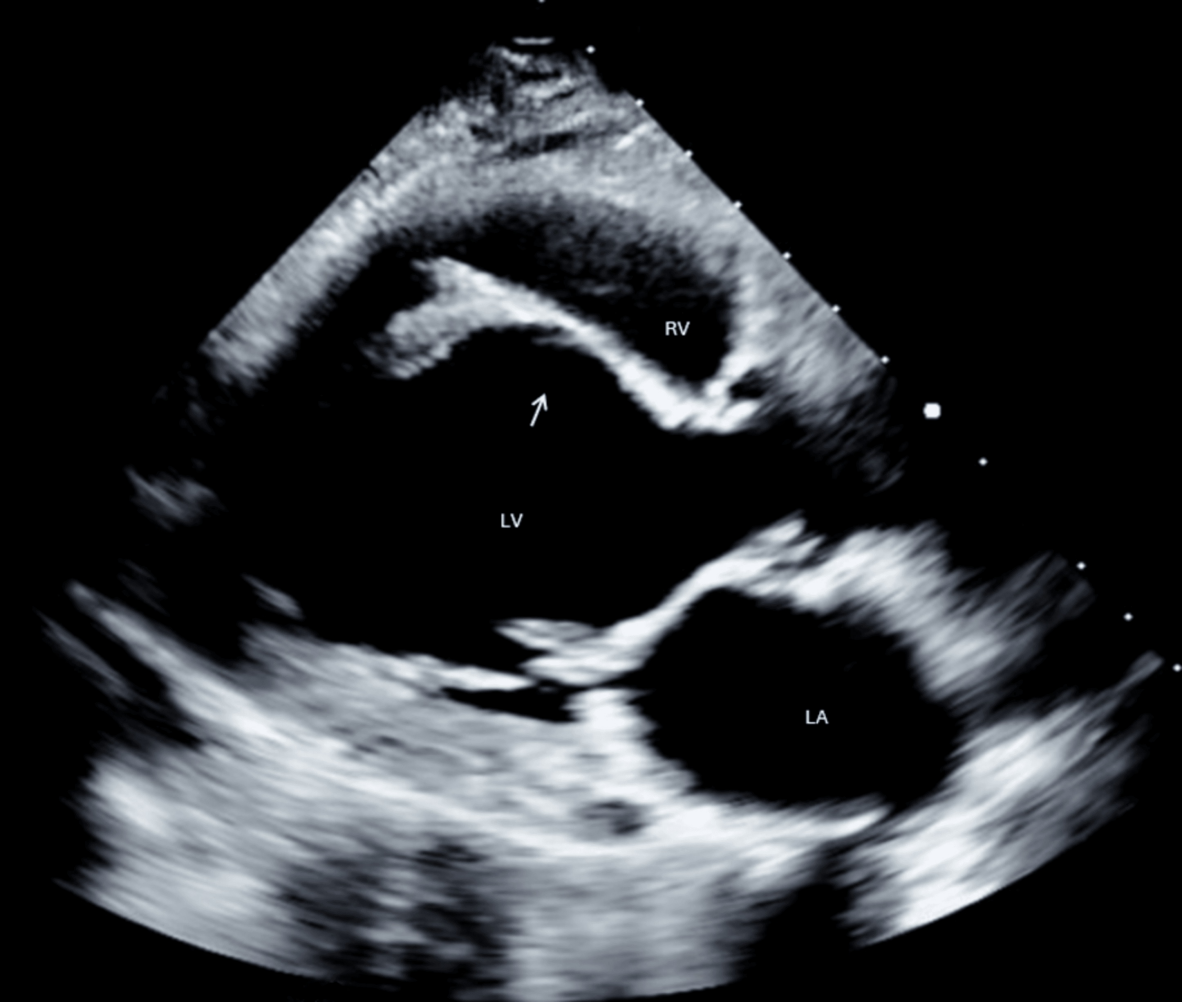
TTE completed approximately four months prior to onset of symptoms. Arrow points to septal aneurysm with bowing into right ventricle during systole. TTE: Transthoracic echocardiogram

A 60-year-old female with multisystem sarcoidosis, including cardiac involvement, presented with sudden-onset palpitations and tachycardia. Her history included heart failure with reduced ejection fraction (HFrEF, 35-40%), a known left ventricular aneurysm, and a recent ventricular tachycardia (VT) storm treated with ablations (six months prior, her third ablation) and an implantable cardioverter-defibrillator (ICD). The patient denied chest pain, dyspnea, and syncope. A transthoracic echocardiogram (TTE) completed approximately four months prior to arrival showed a known ventricular septal wall aneurysm that was first identified four years earlier (Figure 1). The patient was wearing a smartwatch that clearly delineated a sudden onset of tachycardia from her prior resting rate correlating with the onset of symptoms.

Physical examination, labs, and imaging

The patient's vital signs included a blood pressure of 128/93 mmHg, a heart rate of 103 bpm, respiratory rate of 20 breaths per minute, and an oxygen saturation of 98% on room air. On initial examination, the patient appeared to be in mild distress. Cardiovascular assessment revealed tachycardia with a regular rhythm and a newly noted loud 5/6 holosystolic murmur radiating to the carotids. Pulmonary examination demonstrated normal respiratory effort and clear lung fields. Relevant laboratory findings include a troponin I level of 0.03 ng/mL and an elevated brain natriuretic peptide (BNP) of 848 pg/mL (normal <100 pg/mL). Chest X-ray showed stable ICD placement with no acute abnormalities.

POCUS technique and findings

**Figure 2:**
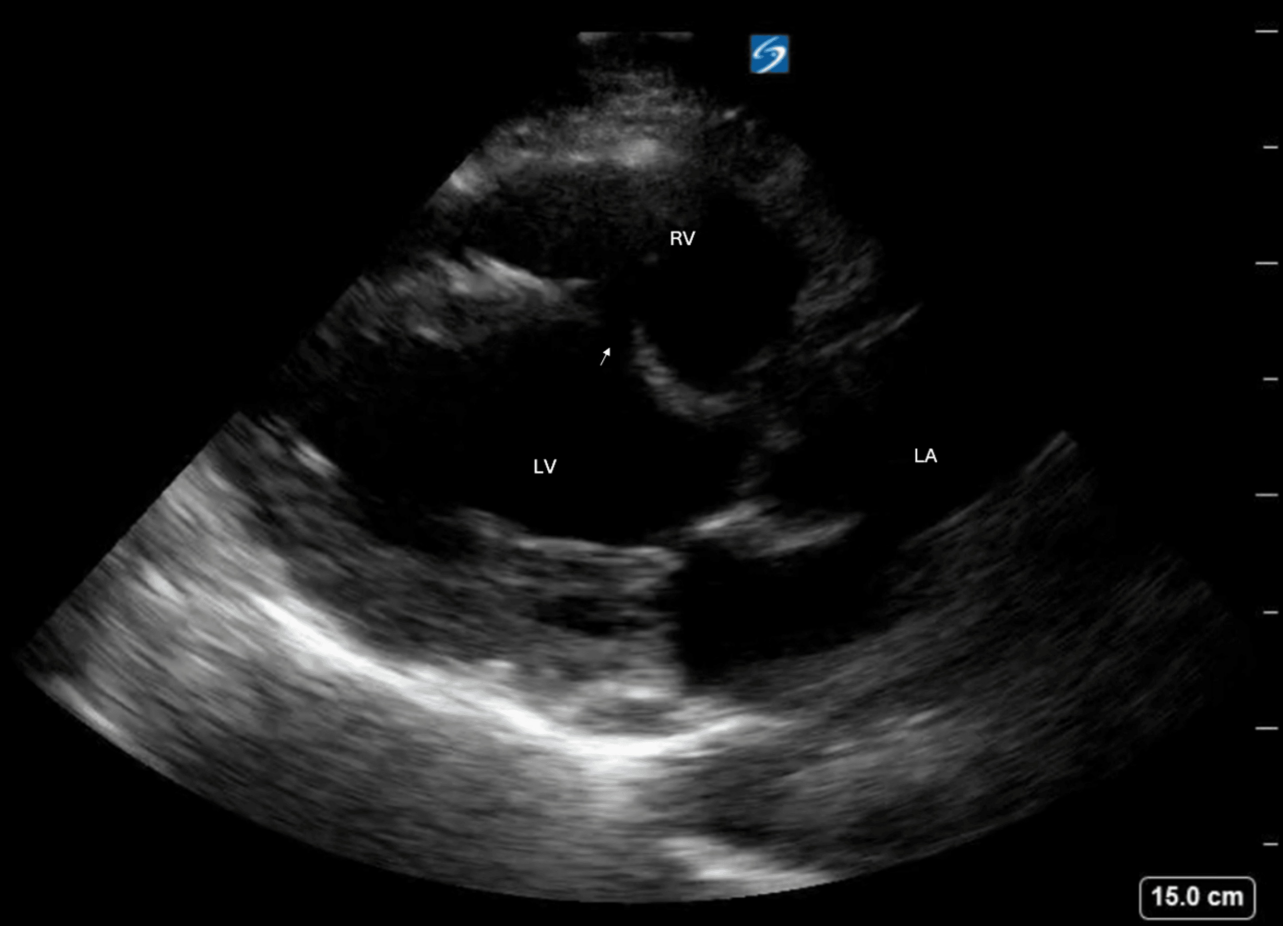
Parasternal long axis view with rupture of ventricular septum with arrow indicating VSR VSR: Ventricular septal rupture

**Figure 3:**
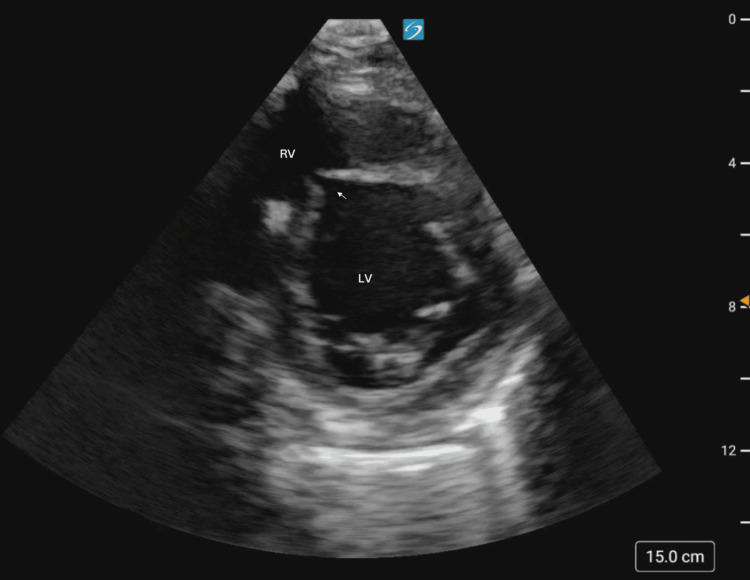
Parasternal short axis view, redemonstration of ventricular septal defect with arrow indicating VSR VSR: Ventricular septal rupture

**Figure 4:**
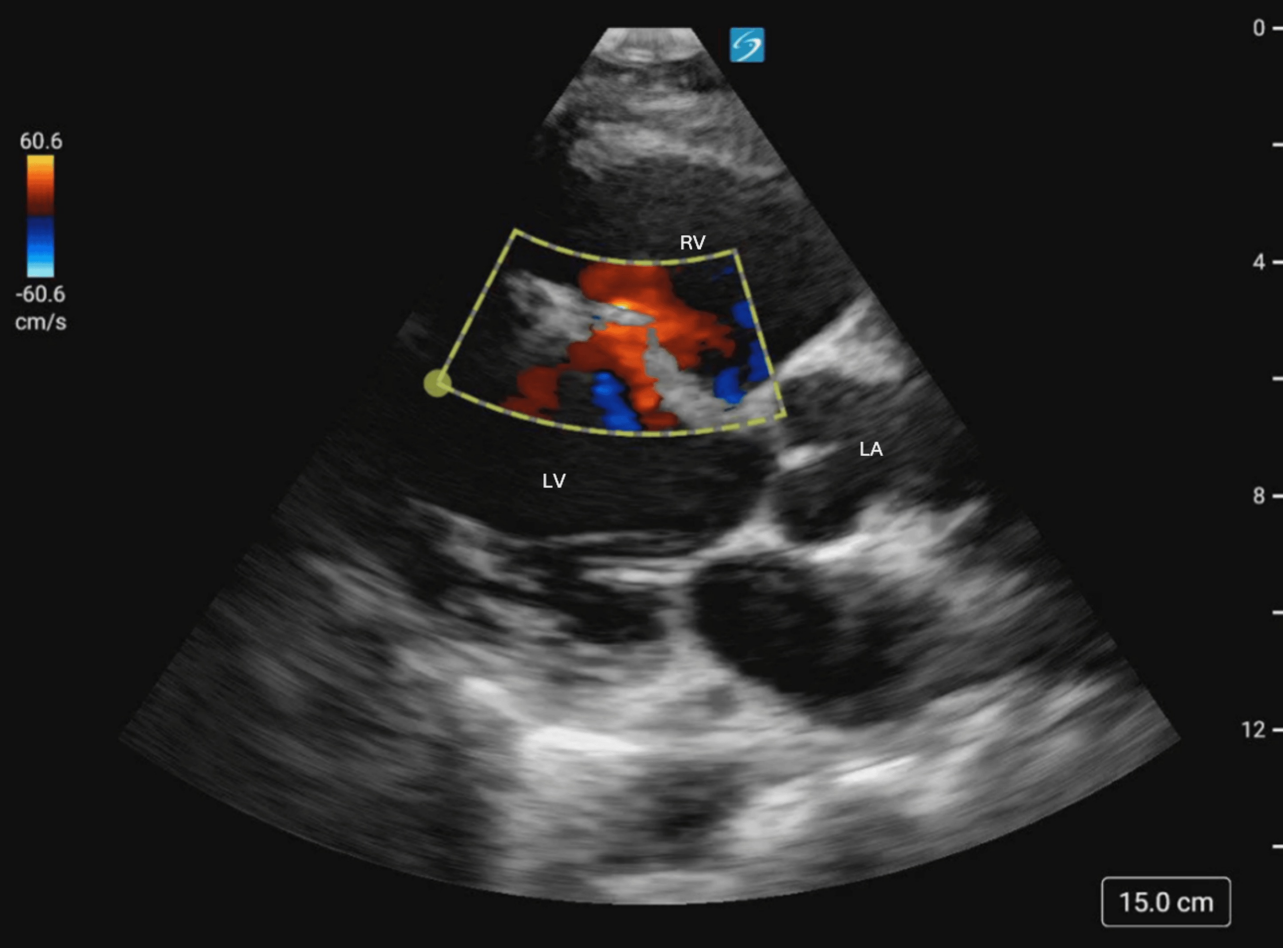
Parasternal long axis view with color doppler with turbulent left to right flow across the ventricular septal wall

**Figure 5:**
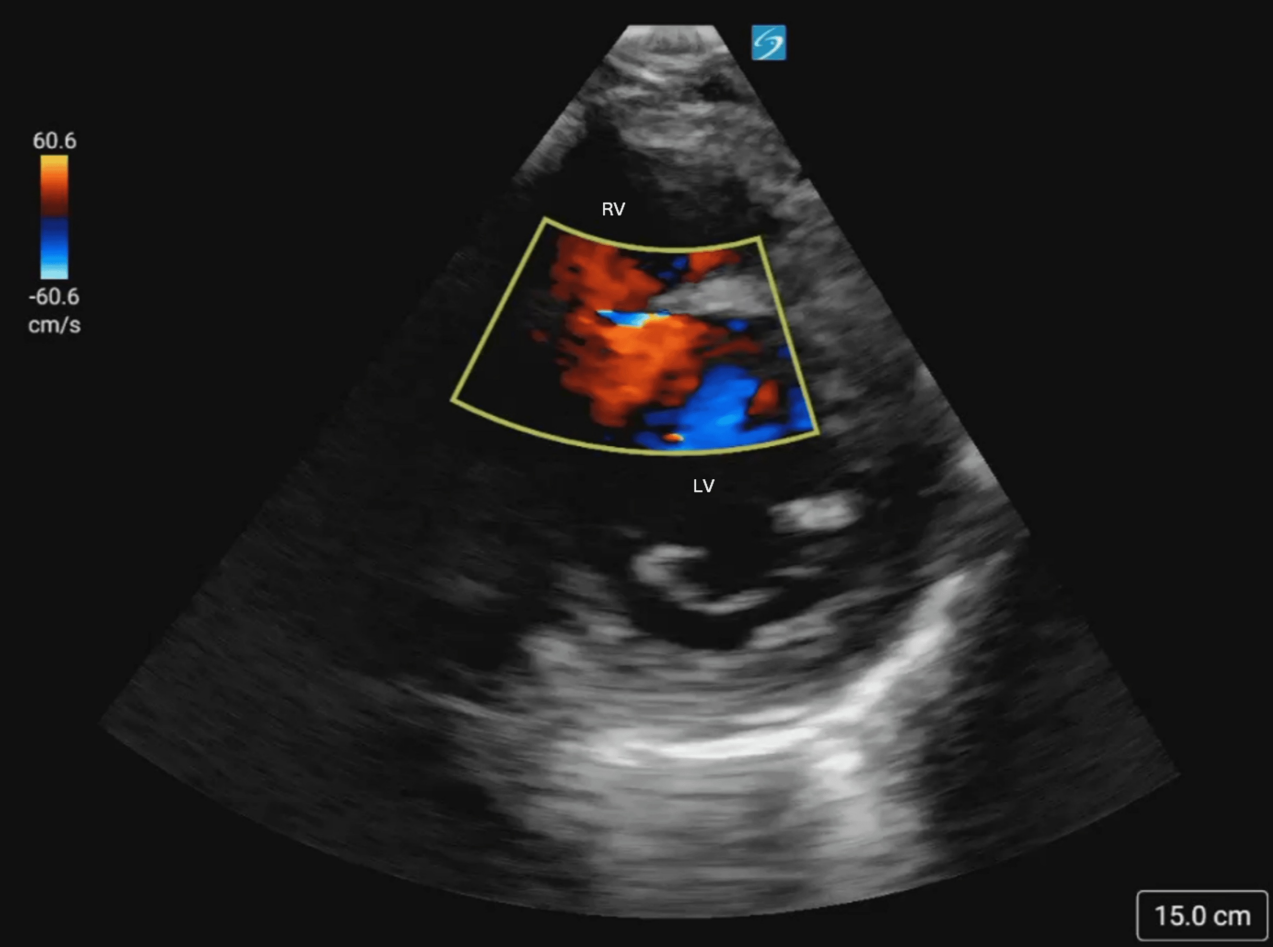
Parasternal short axis with color doppler view showing additional view with turbulent flow across septal wall

Cardiac windows were obtained in both the parasternal long (Figure 2) and parasternal short (Figure 3) axis, demonstrating the new VSR as compared to the prior aneurysm. Color Doppler (Figures 4, 5) demonstrated turbulent trans-septal flow in both parasternal views, confirming the presence of the VSR and highly suggestive of left-to-right shunt.

Clinical course

The patient’s VSR was first identified at a resource-limited free standing emergency department. Despite mild tachycardia, the patient was hemodynamically stable. Cardiology consultation facilitated transfer to a tertiary center via ambulance. A TTE was completed along with cardiac MRI followed by right sided heart catheterization that further clarified the size of the VSR and extent of the shunt. The patient remained stable and was ultimately admitted to the general cardiac floor. The patient was quickly listed for heart transplant after input from cardiac surgery. After an unusually short duration, transplantation was completed on day 16. After a short stay in the cardiac ICU, the patient was eventually discharged home in good health and is resuming her previous quality of life.

## Discussion

VSR and sarcoidosis

Cardiac sarcoidosis is characterized by granulomatous inflammation that can lead to myocardial fibrosis, arrhythmias, and structural abnormalities, including ventricular aneurysms [2,3]. Septal aneurysmal changes are well documented findings in the setting of cardiac sarcoidosis though it is unclear through literature review the risk this poses for VSR. Sato et al. speculated that multiple ablations may increase risk for VSR in sarcoidosis in a patient with septal perforation and progressively worsening heart failure [4]. The clinical presentation of VSR caused by sudden rupture varies based on the size of the defect and the rate of pressure equalization between the left and right ventricles. Patients can present with acute hemodynamic instability, including tachycardia, hypotension, and shortness of breath. The hallmark finding of a VSR is a loud, harsh, holosystolic murmur, often best heard at the left sternal border. In this case, the new onset murmur prompted the initial evaluation. 

VSR often precipitates a sudden left-to-right shunt, which can lead to profound physiological changes. The rapid increase in pulmonary blood flow results in elevated pulmonary venous pressures and subsequent pulmonary congestion or flash pulmonary edema especially with large defects. This can manifest as acute dyspnea, hypoxemia, and tachypnea. Simultaneously, the diversion of blood away from systemic circulation causes a significant drop in cardiac output, leading to hypotension and signs of cardiogenic shock [5].

Management strategies

Acute VSR should be considered a surgical emergency and will require closure to repair the defect, or as in this case transplantation. Stabilizing cardiogenic shock and correcting hypoxia with medical management should be viewed as temporizing measures while plans for definitive care are made. Hemodynamic stabilization may include inotropes and vasodilators, which can reduce afterload and enhance contractility in severe and decompensated cases. Decreasing left ventricle (LV) afterload rapidly should be part of early management in the setting of cardiogenic shock. Nitroglycerine or nitroprusside can reduce LV afterload with the goal to minimize blood shunted across the ventricular defect [6]. For unstable patients, preoperative stabilization may require intra-aortic balloon pumps (IABPs), extracorporeal membrane oxygenation (ECMO), or Impella devices to support circulation.

Oxygenation strategies may present unique challenges and require a nuanced approach. Simple oxygenation supplementation may be enough in the setting of mild hypoxia, however with the occurrence of flash pulmonary edema, positive pressure management may be indicated. This escalation needs to be carefully monitored as the decreased venous return may further exacerbate cardiac output and worsen symptoms of cardiogenic shock [5].

Sarcoidosis may pose additional challenges due to its potential for multi-organ involvement, including simultaneous cardiac and pulmonary diseases. There may be rare instances of both pulmonary and cardiac sarcoidosis that may further complicate decision making and management. In addition to the VSR, these patients may be at higher risk of pre-existing pulmonary hypertension, increasing the risk of right-to-left shunting [6].

Most documented cases of VSR follow a prior ischemic event and can be observed in as little as one week after ischemic injury. While VSR in the setting of cardiac sarcoidosis is exceptionally rare, new onset murmur and hemodynamic changes should raise concern for potentially catastrophic changes. Regardless of the underlying cause, management remains similar, as does the presentation, exam, and ability of POCUS to provide rapid diagnostic information [5,7]. 

POCUS utility and diagnostic value

New onset murmurs should raise concern for new and life-threatening pathology. Identifying the timing in the cardiac cycle is key to narrowing the differential and understanding potential pathology [8]. The timing, grade, radiation, and associated hemodynamic changes of a new holosystolic murmur may all provide clues to the underlying cause; together, auscultation and POCUS can provide critical and timely diagnostic imaging to differentiate the possible causes [9].

POCUS facilitated the rapid and accurate identification of the VSR, expediting diagnosis and management. The rapid detection of structural abnormalities and turbulent flow demonstrated the value of bedside imaging in resource-limited settings and expedited steps for proper disposition and definitive care.

## Conclusions

This case illustrates the critical role of POCUS in diagnosing life-threatening complications of cardiac sarcoidosis. Emergency physicians must maintain a high index of suspicion for structural cardiac abnormalities in patients with complex cardiac histories and utilize POCUS to guide early intervention and improve outcomes. By leveraging POCUS, clinicians can bridge diagnostic gaps in resource-limited settings and deliver timely, life-saving care. In this case, POCUS provided immediate and actionable diagnostic clarity, significantly impacting the patient’s disposition and management. POCUS, as an accessible and dynamic imaging tool, underscores its importance in emergency settings where new onset heart murmurs are present.

## References

[1] Cheng RK, Kittleson MM, Beavers CJ (2024). Diagnosis and management of cardiac sarcoidosis: a scientific statement from the American Heart Association. Circulation.

[2] Uchida M, Shinohara T, Takahashi N, Saikawa T (2012). Interventricular septal mass in a patient with cardiac sarcoidosis. J Cardiovasc Electrophysiol.

[3] Jmeian A, Thawabi M, Goldfarb I, Shamoon F (2015). Left ventricular aneurysm and ventricular tachycardia as initial presentation of cardiac sarcoidosis. N Am J Med Sci.

[4] Sato K, Kawamatsu N, Kimata A, Matsubara M, Ieda M (2022). Cardiac sarcoidosis complicated by ventricular septal perforation after multiple radiofrequency ablations for ventricular tachycardia. Eur Heart J Cardiovasc Imaging.

[5] Novak M, Hlinomaz O, Groch L, Rezek M, Semenka J, Sikora J, Sitar J (2015). Ventricular septal rupture—a critical condition as a complication of acute myocardial infarction. J Crit Care Med (Targu Mures).

[6] Alviar CL, Miller PE, McAreavey D (2018). Positive pressure ventilation in the cardiac intensive care unit. J Am Coll Cardiol.

[7] Kirbos C, Pagenhardt J, Minardi J, End B (2022). Point-of-care ultrasound diagnosis of ventricular septal rupture post myocardial infarction: a case report. J Emerg Med.

[8] Landefeld J, Tran-Reina M, Henderson M (2022). Approach to the patient with a murmur. Med Clin North Am.

[9] Attenhofer Jost CH, Turina J (2000). Echocardiography in the evaluation of systolic murmurs of unknown cause*. Am J Med.

